# Enriched expression of genes associated with autism spectrum disorders in human inhibitory neurons

**DOI:** 10.1038/s41398-017-0058-6

**Published:** 2018-01-10

**Authors:** Ping Wang, Dejian Zhao, Herbert M. Lachman, Deyou Zheng

**Affiliations:** 1 Department of Genetics, Albert Einstein College of Medicine, 1300 Morris Park Ave., Bronx, NY USA; 2 Department of Psychiatry and Behavioral Sciences, Albert Einstein College of Medicine, 1300 Morris Park Ave., Bronx, NY USA; 3 Department of Neuroscience, Albert Einstein College of Medicine, 1300 Morris Park Ave., Bronx, NY USA; 4 Department of Medicine, Albert Einstein College of Medicine, 1300 Morris Park Ave., Bronx, NY USA; 5 Department of Neurology, Albert Einstein College of Medicine, 1300 Morris Park Ave., Bronx, NY USA

## Abstract

Autism spectrum disorder (ASD) is highly heritable but genetically heterogeneous. The affected neural circuits and cell types remain unclear and may vary at different developmental stages. By analyzing multiple sets of human single cell transcriptome profiles, we found that ASD candidates showed relatively enriched gene expression in neurons, especially in inhibitory neurons. ASD candidates were also more likely to be the hubs of the co-expression gene module that is highly expressed in inhibitory neurons, a feature not detected for excitatory neurons. In addition, we found that upregulated genes in multiple ASD cortex samples were enriched with genes highly expressed in inhibitory neurons, suggesting a potential increase of inhibitory neurons and an imbalance in the ratio between excitatory and inhibitory neurons in ASD brains. Furthermore, the downstream targets of several ASD candidates, such as *CHD8*, *EHMT1* and *SATB2*, also displayed enriched expression in inhibitory neurons. Taken together, our analyses of single cell transcriptomic data suggest that inhibitory neurons may be a major neuron subtype affected by the disruption of ASD gene networks, providing single cell functional evidence to support the excitatory/inhibitory (E/I) imbalance hypothesis.

## Introduction

Autism spectrum disorder (ASD) is a class of neurodevelopmental disorders characterized by persistent deficits in social communication/interaction and restricted, repetitive patterns of behaviors, interests or activities (DSM-5)^1^. Recent epidemiology studies have reported that 1 in 68 children is diagnosed with ASD, with a 3 to 4-fold increased risk for boys^2,3^. Family and twin studies have found that ASD is highly heritable^4,5^, but the genetic risk factors for ASD are highly heterogeneous and up to one thousand genes are estimated to be involved, with no single gene accounting for >1–2% of the cases^6^. These ASD candidate genes converge on several molecular and cellular pathways, such as synaptic function, Wnt-signal and chromatin remodeling^7–12^, indicating that ASD pathogenesis is a complicated multidimensional process modulated by genetic factors that play key roles in response to intrinsic developmental signaling and environmental perturbations.

At the cellular level, a human brain can be divided into distinct functional regions that are composed of diverse but densely connected cell types. It has been reported that ASD risk genes form co-expression networks that are expressed at relatively higher levels in specific embryonic prefrontal cortex regions and layers^13,14^, and ASD mutations could potentially affect certain brain areas and cell types more strongly than others^15^. For example, Xu et al. previously developed a method (“cell type-specific expression analysis”) to analyze microarray gene expression data from mouse and human brains, including cell type data from translating ribosome affinity purification (TRAP) technology, and found that multiple cell types could be implicated in ASD^16^, e.g., astrocytes, glia and cortical interneurons. Subsequently, Zhang et al., also using TRAP data from mouse lines, observed that an expression signature shared by ASD risk genes is a strong and positive association with specific neurons in different brain regions, including cortical neurons^17^.

A limitation of these previous studies is related to the concern that the resolution of cell types may not be sufficient, in addition to other limitations specifically related to the microarray platform. This can be addressed by single cell RNA-seq (scRNA-seq) analysis that measures gene expression profiles for hundreds to thousands of cells in a tissue sample simultaneously, which can resolve cell types and reveal expression heterogeneity^18^. With a mouse scRNA-seq dataset^19^ and a novel computational method, Skene et al. suggested that genetic susceptibility of ASD primarily affected interneurons and pyramidal neurons^20^. The method is called “expression weighted cell-type enrichment” (EWCE), which evaluates statistically whether a set of genes shows higher expression in a particular cell type than what is expected by chance^20^. Skene et al recently also reported that pyramidal cells, medium spiny neurons and certain interneurons could be more important for schizophrenia than other brain cell types^21^.

While the above studies have suggested that ASD risk genes can have cell type specific functions and expression patterns, and some brain cell types may be more prone to the effects of ASD-associated mutations, no similar studies have been performed using human cell type-specific functional genomic data, especially scRNA-seq data. This is important because it has been shown that many gene expression modules are human specific, several of which are correlated with brain disorders, such as Alzheimer’s disease^22^, despite the extensive global network similarity of the human and mouse brain transcriptomes. Our previous study of the transcriptional regulatory network modulated by a neural master regulator, REST/NRSF, also found that ASD genes are enriched among human specific REST targets^23^. Moreover, the human brain is much more complex than the mouse brain, especially in some regions, such as the frontal and temporal lobes, which have undergone enormous changes during primate evolution^24^. More importantly, no systematic studies related to ASD have been carried out in which excitatory and inhibitory neuronal transcriptomes have been compared, despite the long-standing E/I imbalance hypothesis, which has been proposed as a model to explain some ASD-related behaviors^25–28^. Therefore, to address if some cell types are more prone to genetic network disruptions potentially occurring in the brains of individuals with ASD, we have collected multiple human neural or brain expression datasets, most of which were derived from advanced scRNA-seq analysis, and evaluated if genes implicated in ASD show different expression profiles across human neural cell types. The gene sets in our study include a) ASD candidate genes, b) differentially expressed genes between ASD individuals and controls, and c) downstream targets of several ASD candidates. We found that these genes consistently show significantly enriched expression in human neurons, particularly inhibitory neuron, suggesting that inhibitory neuron is the major cell type affected in ASD. This finding is consistent with the hypothesis that a disruption of the balance between inhibitory and excitatory signaling could be an important underlying mechanism of ASD pathogenesis.

## Materials and methods

### Human single cell RNA-seq data

Four sets of human scRNA-seq data were analyzed. For the fetal brain and cerebral organoid datasets^29^ and the adult brain dataset^30^, raw scRNA-seq reads were aligned to the human reference genome (GRCh37/hg19) using STAR (ver. 2.0.13)^31^. Duplicate reads were removed using Samtools (ver. 0.1.19)^32,33^. Gene length and uniquely mapped reads for each gene were calculated using featureCounts in subread package (ver. 1.4.6)^34^ with gene models from Ensembl release 74. Fragments per kilobase of transcript per million mapped reads (FPKM) values were calculated using R (https://www.r-project.org/) according to its definition. For the neuron subtype dataset for excitatory and inhibitory neurons, transcripts per kilobase million (TPMs) were obtained from the original paper^35^. In all four cases, we used the authors’ original classification of cell types.

### Lists of genes associated with ASD, schizophrenia and other brain disorders

ASD candidate genes were downloaded from the SFARI database (https://gene.sfari.org/autdb/GS_Home.do; genes scored as high confidence, to minimal evidence and syndromic) and the AutismKB (core dataset)^36^. The two schizophrenia gene lists were from the SZgene database^37^ and a recent GWAS report^38^. Bipolar disorder associated genes were from the BDgene database^39^. Other gene lists associated with brain diseases were described in our previous publication^23^. Genes encoding excitatory and inhibitory postsynaptic density (PSD) proteins were from a previous study by Uezu et al.^40^ The genes associated with human height were from a previous GWAS^41^. Gene lists were provided in Table S1.

### Differentially expressed genes between ASD and controls

Gene expression in postmortem cortices (“Cortex1”)^42^ was used to detect differentially expressed genes by GEO2R (https://www.ncbi.nlm.nih.gov/geo/geo2r/), which is defined here as FDR < 0.05 and fold change > 1.3—the same criteria as used in the original paper. Differentially expressed genes in blood^43^ were also detected by GEO2R and defined as *p* < 0.05. Gene lists from other brain-related samples, including postmortem cortices (“Cortex2”^44^ and “Cortex3”^45^), induced pluripotent stem cell (iPSC)-derived cerebral organoids (“Organoid”)^46^, neural progenitor cells (NPC)^47^, and neurons (“Neuron1”^47^ and “Neuron2”^48^), were obtained from the original papers and provided in Table S1.

### Downstream genes of ASD candidates

CHD8-regulated genes in NPCs, neurons^49^ and cerebral organoids^50^, CYFIP1-regulated genes^51^, TCF4 and EHMT1-regulated genes^52^, MBD5 and SATB2-regulated genes^53^, NRXN1-regulated genes^54^ and ZNF804A-regulated genes^55^ were from studies where the expression of a known ASD candidate was reduced by knockout or knockdown. Gene lists were obtained from the original papers and provided in Table S1.

### EWCE analysis for determining enriched expression

For each cell type, mean of log2(FPKM or TPM) across all samples were calculated and imported into the EWCE (v1.3.0)^20^ to determine enriched expression. To determine specifically which genes contributed to the statistical significance of enriched expression, we used the “generate.bootstrap.plots” function in EWCE with 10,000 permutations to generate “mean bootstrap expression” (see Fig. 2 in the EWCE paper^20^ for details). We further defined genes whose relative expression was >1.2-fold greater than the mean bootstrap expression as “enriched genes”. The resultant lists of enriched genes were analyzed for enriched gene ontology (FDR < 0.05) using the software goseq^56^, in which the corresponding full gene list was used as background.

### Weighted gene co-expression network analysis (WGCNA)

Signed co-expression networks were built using the WGCNA package^57^. The power of 18 was chosen, and blockwiseModules function was performed to build networks. Logistic regression was used to find modules expressed higher in excitatory or inhibitory neurons using eigengenes. P values were corrected by multiple testing to generate FDR. ToppGene^58^ was used to find Gene Ontology categories enriched in modules.

### Code availability

The primary software EWCE (v1.3.0)^20^ was downloaded from https://www.bioconductor.org/packages/release/bioc/html/EWCE.html. Additional codes for data processing are available from the authors upon request. An early version of this manuscript^59^ was submitted to bioRxiv before publication.

## Results

### ASD candidate genes show enriched expression in neurons, especially inhibitory neurons

**Fig. 1:**
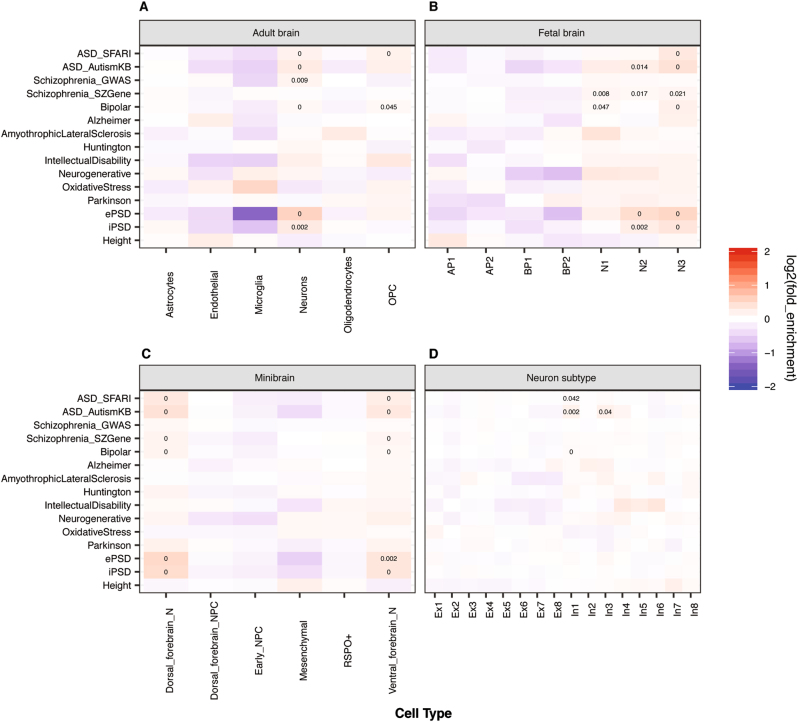
Cell type enrichment analysis of genes associated with ASD or other brain disorders across multiple single cell transcriptome datasets. **a** Adult brains, **b** Fetal brains, **c** Cerebral organoids, and **d** Neuron subtypes. The color in each panel represents fold enrichment, calculated as the expression of the target gene lists divided by the mean expression of the randomly selected genes in bootstrap sampling by EWCE. The number in individual boxes represents significant adjusted p value (FDR < 0.05). *ePSD* excitatory postsynaptic density, *iPSD* inhibitory postsynaptic density, *OPC* oligodendrocyte precursor cell, *AP* apical progenitor, *BP* basal progenitor, *N* neuron, *NPC* neural progenitor cells, *Ex* excitatory neuron, *In* inhibitory neuron. The same annotations are used for the colors and numbers of the boxes in Figs 3 and 4 below

It has generally been supposed that functional disruptions of a gene more likely affect the cells or tissues where the gene is highly expressed. Such a principle has often been used to support the discovery of risk genes from genetic studies in schizophrenia and ASD^38,60^. Accordingly, we have used the EWCE method to test what brain cell types are more likely to be affected by genes implicated in ASD, using transcriptomic data containing cell type identifies. Throughout this paper, the term “enrichment expression” or “enriched expression” in a particular cell type refers to a set of genes that have a higher level of expression within this cell type than expected by chance, as described in the EWCE method^20^. The method also accounts for a gene’s overall expression across all cell types in a comparison, as the enrichment is actually computed based on relative expression values. We started with scRNA-seq expression data from six cell types from adult human brains (21–63 years old; a total of 285 cells), including neurons, microglia, and astrocytes (Fig. 1a)^30^. First, as a negative control, we found that genes associated with human height^41^ showed no enrichment of expression in any of the six cell types in test (Fig. 1). Conversely, as a positive control, genes encoding PSD proteins showed significant enrichment in neuron expression (Fig. 1).

Our analysis of the ASD candidates, obtained from either the SFARI (https://gene.sfari.org/autdb/GS_Home.do) or the AutismKB^36^, demonstrated that their expression was significantly enriched in human adult neurons and oligodendrocyte precursor cells but not astrocytes and microglia (Fig. 1a). Note that the significance of enrichment was a result of many but not few genes (Fig. S1), an observation applicable to results described below too (Table S2). As ASD is an early developmental disorder, we repeated the same analysis using a single cell transcriptome dataset from human fetal brains, including 226 single-cell transcriptomes from 12 and 13-wk post-conception neocortex specimens^29^. The cell types in the fetal brain were classified differently from those in adult brains. We found that in comparison to apical and basal progenitors, ASD candidates were significantly enriched in neurons, especially mature neurons (“N2” and “N3”) in fetal brains (Fig. 1b). We also found schizophrenia and bipolar disorder associated genes were similarly enriched in mature neurons (Fig. 1b), consistent with the known overlap of genetic risk factors among these disorders^61^. Meanwhile, genes associated with several other brain diseases, such as Alzheimer and Huntington, showed no significantly enriched expression in any of these cell types (Fig. 1b). Next, to study whether ASD candidates are enriched in neurons in specific brain regions, we analyzed single cell transcriptome data of cerebral organoids, including 495 single-cell transcriptomes^29^. Again, compared with NPCs, ASD candidates displayed significantly enriched expression in neurons - both dorsal and ventral forebrain neurons, as were schizophrenia and bipolar disorder associated genes (Fig. 1c). While not quite surprising, our analysis of these three cell type transcriptomic datasets showed that neurons, both early fetal neurons and adult neurons, are a major cell type potentially affected by ASD mutations, probably more so than neural progenitors. Finally, neurons could be largely classified into two major subtypes: excitatory and inhibitory neurons. Using scRNA-seq data of neuronal subtypes, whose classification was supported by known marker genes and including 3083 single-cell transcriptomes from six cortical regions of a control normal 51-year-old female postmortem brain^35^, we found that the expression of ASD candidates was significantly enriched in inhibitory neurons, especially among the subtypes “In1” and “In3” (Fig. 1d), which are superficial layer inhibitory neurons that originate from lateral ganglionic eminences^35^. These results suggest that functional disruptions of ASD genes as a group can affect inhibitory neurons more than excitatory neurons. Again, the significant enrichments were results from many genes (Fig. S2). For ASD genes, the expression “enriched genes” (see Methods) include neuronal markers, such as *GAD1*, *RELN*, *VIP*, as expected, and transcription regulators, such as *CHD7*, *PAX6* and *TBX1* (Table S2). Although it remains to be established with functional assays, this finding indicates that inhibitory neuron transcriptome dysregulation can occur in ASD brains, which is consistent with the E/I imbalance hypothesis in ASD^25,62–65^. GABAergic neurotransmission appears to play a role in both schizophrenia and bipolar disorder as well^66,67^. However, our results suggest that bipolar disorder but not schizophrenia-associated genes were significantly enriched among highly expressing genes in inhibitory neurons.

### ASD candidate genes are more likely to be hubs of co-expression modules in inhibitory neurons

**Fig. 2:**
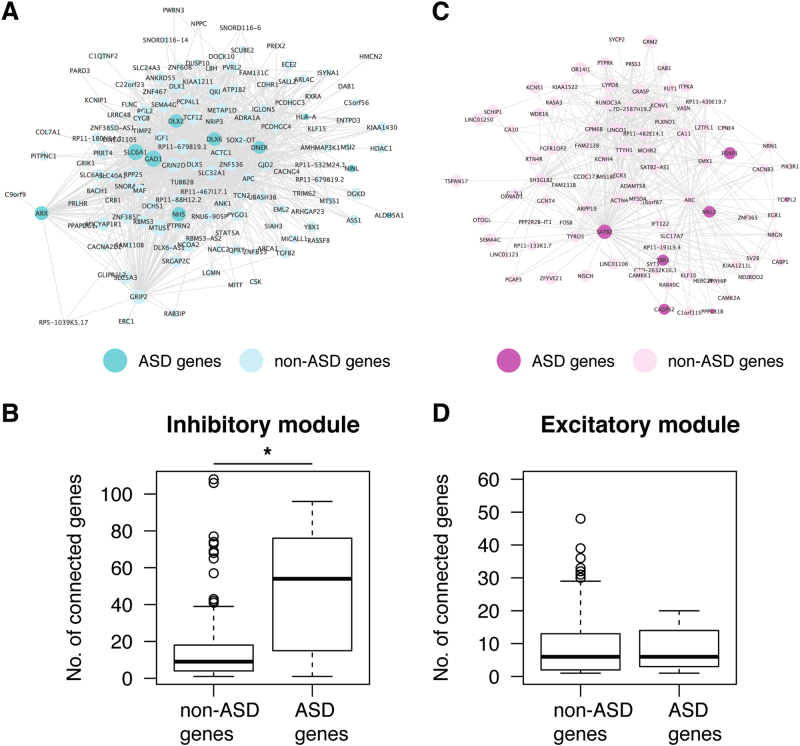
Visualization of gene co-expression module associated with excitatory and inhibitory neurons. Genes with co-expression coefficient > 0.2 from the WGCNA are shown as a network for **a** inhibitory and **c** excitatory modules. Node size represents the number of connected genes. Darker nodes are ASD candidate genes. Boxplots show the number of connections for ASD and non-ASD genes in inhibitory **b** and excitatory **d** modules. * p<0.05 Wilcoxon test

To further study the roles of ASD candidate genes in inhibitory neurons, we performed WGCNA to build a co-expression network from the neural subtype transcriptome data^35^, resulting in 73 modules (Figure S3A and B). One of them showed high expression in excitatory neurons and contained 1936 genes, which were enriched for functions related to synaptic signaling, neuron projection and morphogenesis, as well as genes expressed in excitatory synapses (Figure S3,C). Another module contained 951 genes that were highly expressed in inhibitory neurons. They were enriched with genes involved in neurogenesis, positive regulation of synaptic transmission, and the GABA shunt (Figure S3,D). Consistent with the EWCE result, ASD candidates, from both the SFARI and AutismKB, were more significantly enriched in the module highly expressed in inhibitory neurons (odds ratio (OR) = 2.38, *p* = 5.94e-07, Fisher’s exact test, one-tailed) than the module highly expressed in excitatory neurons (OR = 1.42, *p* = 0.018, Fisher’s exact test, one-tailed). Among the hub genes in the inhibitory module, nine were ASD candidates (Fig. 2a), including three genes encoding transcription factors (*ARX*, *DLX2,* and *DLX6*) that are important for appropriate migration of inhibitory neurons to the cortex^68^, and three genes (*SLC6A1*, *GAD1*, *ALDH5A1*) that participate in GABA synthesis, release, reuptake and degradation, as described in the Reactome pathway^69^. Notably, those ASD candidate genes had more connections in the inhibitory module than non-ASD candidates (*p* = 0.0057, Wilcoxon test; Fig. 2b), suggesting that ASD candidates tend to be the hubs in inhibitory module, and consequently, disease-associated mutations would likely lead to a disruption of the co-expression network. By comparison, in the excitatory module, ASD and non-ASD candidate genes had similar connections (*p* = 0.72, Wilcoxon test; Fig. 2c, d).

### Genes up-regulated in ASD-derived neuronal samples show enrichment in inhibitory neurons

**Fig. 3:**
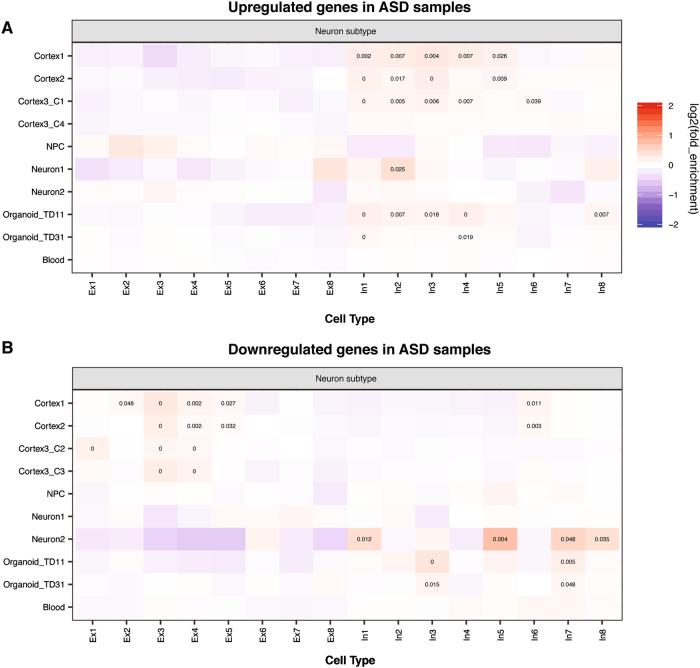
Cell type enrichment analysis of upregulated **a** and downregulated **b** genes in ASD samples. Note that ASD candidates from SFARI or AutismKB have been excluded from DEGs

Because of the extensive genetic heterogeneity in ASD, investigators have carried out transcriptomic studies in postmortem samples or ASD patient-derived neural samples with the goals of finding common pathways and cellular processes dysregulated in ASD brains or neural samples^42–48^. We thus decided to study whether differentially expressed genes (DEGs) in molecular studies carried out between ASD and control subjects exhibited similar cell type-biased expression patterns as ASD candidate genes identified from genetic studies. We obtained DEGs in ASD brain or blood samples and analyzed their expression across brain cell types. Since, as shown above, we had uncovered the biased expression pattern of ASD candidate genes (from SFARI or AutismKB), we excluded them from the DEGs during EWCE analysis, in order to focus on downstream effects. In ASD cortex samples, up-regulated genes were enriched with genes highly expressed in adult astrocytes and microglia (Fig. S4A), whereas down-regulated genes were enriched with genes highly expressed in neurons (Fig. S4B). This is consistent with previous reports^42,44,45^, but extends the finding to relatively mature neurons and both dorsal and ventral forebrain neurons (Fig. S4,B). We also found up-regulated genes in ASD cortex samples were enriched for highly expressed genes in NPCs (Fig. S4,A), a pattern not detected when ASD candidates were analyzed (Fig. 1). However, genes up-regulated in NPCs, neurons and cerebral organoids derived from ASD iPSC-lines showed enriched expression in neurons (Fig. S4,A), while down-regulated genes in the patient-derived samples were enriched with genes expressed highly in astrocytes, microglia and NPCs (Fig. S4,B). These results suggest that cell types can be affected differently in early and late developing ASD brains. The difference may also reflect primary vs secondary effects. However, in our comparison of excitatory vs inhibitory neurons, we found that up-regulated genes in both postmortem cortices and cerebral organoids were similarly enriched with genes highly expressed in inhibitory neurons (Fig. 3a). In a group of upregulated genes in ASD cortices (Cortex3_C1), enriched genes for In4 were enriched with regulation of cell motility; in upregulated genes in ASD-derived organoids (Organoid_TD11), enriched genes for In1 and In4 were enriched with DNA binding (Table S3). The down-regulated genes from cortices and iPSC-derived neurons or cerebral organoids exhibited opposite enrichments, with the former enriched for high expression in excitatory and the latter in inhibitory neurons (Fig. 3b). Importantly, dysregulated genes in ASD blood samples^43^ did not exhibit any significant pattern of expression enrichment.

### Downstream transcriptional targets of key ASD candidates are enriched among genes expressed highly in inhibitory neurons

**Fig. 4:**
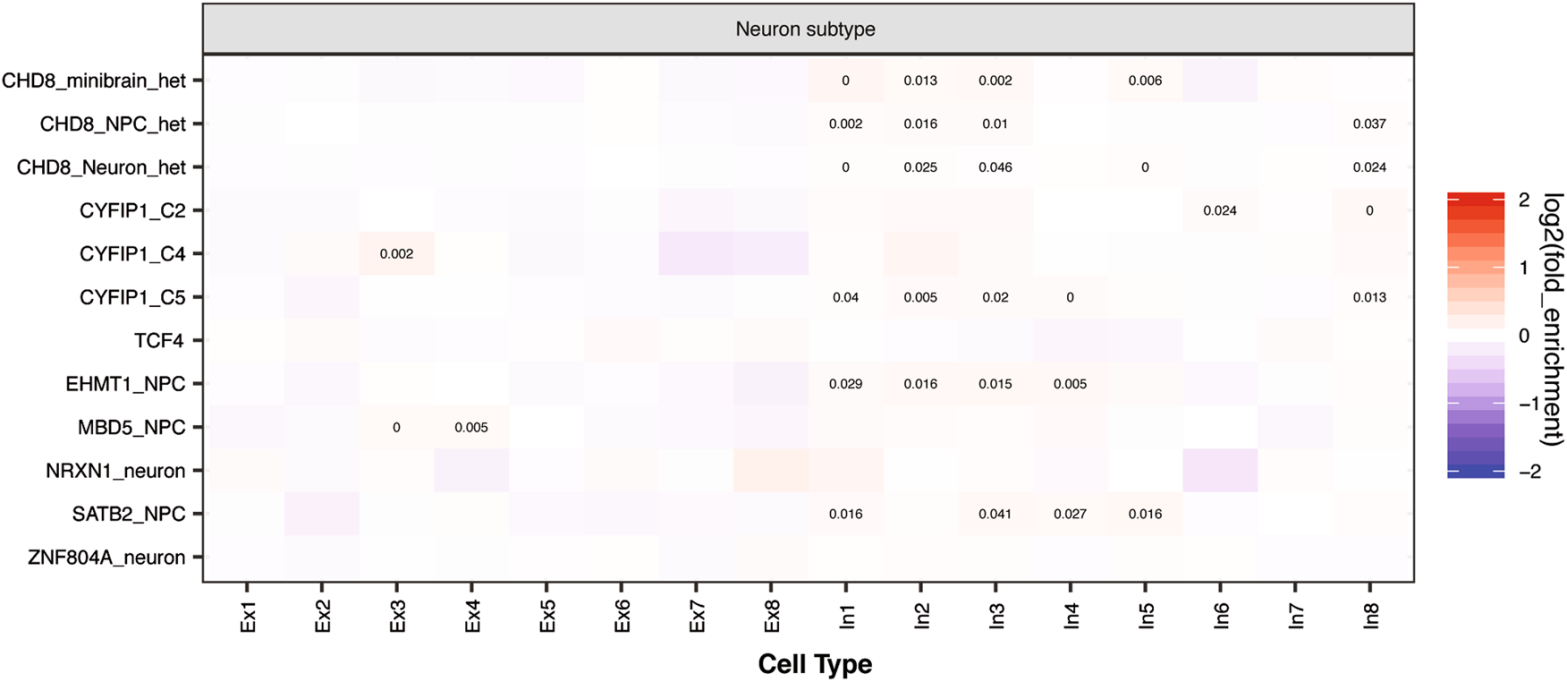
Cell type enrichment analysis of downstream targets of ASD candidates

Finally, we studied whether the downstream targets of ASD candidates genes show different expression enrichment patterns between inhibitory and excitatory neurons by analyzing the DEGs in human neural samples in which the expression of several top ASD (or schizophrenia) candidate genes have been reduced by either knockout or knockdown. We found that CHD8, EHMT1 and SATB2 regulated genes were exclusively enriched in inhibitory neurons (Fig. 4). For CHD8 regulated genes in iPSC derived neurons, expressionally enriched genes for In1/2/3/8 were functionally enriched for single-multicellular organism process; for SATB2 regulated genes, enriched genes were enriched with embryonic organ development; for CYFIP1 regulated genes, enriched genes for In2 and In4 are enriched with cell differentiation and locomotion, respectively (Table S3). Moreover, a general enrichment in inhibitory neuronal genes, especially those in “In1/2/3” classes, was found among the targets of ASD candidates (Fig. 4). Among the downstream targets, *DLX1*, a transcription factor critical for inhibitory neuron function is markedly upregulated in ASD patient-derived telencephalic organoids^46^ and *CHD8* knockout cerebral organoids^50^, but *GAD1*, an inhibitory neuron marker, was downregulated in *SATB2* knockdown samples^53^. We analyzed DEGs from *CYFIP1* knockdown in NPCs derived from three independent iPSC-lines and found both common and distinct enriched expression patterns. DEGs from two lines (C2 and C5) were enriched in inhibitory neurons, but C4 DEGs showed enriched expression in excitatory neurons (Fig. 4). This difference could reflect the limited overlap of the DEGs^51^, but also suggests an intriguing possibility that E/I imbalances could be affected by inter-individual differences in genetic background. We should point out that *CHD8* and *EHMT1* are expressed at a similar level in excitatory and inhibitory neurons, but *SATB2* is expressed at a higher level in excitatory neurons. These findings further suggest that some ASD genes can affect the expression of key genes important for inhibitory and excitatory neurons and their targets may be involved in the interaction or signaling balance between the two types of neurons.

## Discussion

By integrating ASD candidates, dysregulated genes in ASD samples and downstream targets of ASD candidates with recently published human scRNA-seq datasets, we found that ASD-associated genes exhibited enriched expression in neurons, especially inhibitory neurons, with some developmental stage differences. The enrichment of inhibitory neuronal expression among ASD candidate genes provides molecular support for the finding that deficits in inhibitory neuronal function occurs in some syndromes with autism-associated behaviors, such as individuals with *ARX* mutations^70,71^, Dravet syndrome caused by loss-of-function mutations in *SCN1A* ^72^, and Tuberous Sclerosis caused by mutations in *TSC1/2* ^73,74^ (for review, see ref. ^75^). Our current findings are in line with the long-standing hypothesis that E/I signaling imbalance contributes to ASD. The attractive theory of an increase in the ratio between excitatory and inhibitory signaling provides a plausible explanation for the relative reduction in GABAergic signaling found in patients with ASD and their propensity to develop epilepsy^75^. However, a relative excess of inhibitory neuronal activity has been observed in mouse models of Rett Syndrome^76^, and mice with a targeted *Mecp2* deletion restricted to GABAergic inhibitory neurons recapitulates most of the ASD-like features observed in animal models^77^, while restoring *Mecp2* expression reverses some of the phenotypical defects^78,79^.

Our analysis showed enriched expression in inhibitory neurons for upregulated but not down-regulated genes in ASD samples. This seems inconsistent with the enriched expression of ASD candidates in inhibitory neurons, assuming their mutations lead to reduced expression and functional loss. One possibility is that some ASD candidates may function as transcriptional inhibitors or the abnormal expression of some ASD candidates could lead to an increase in the number of inhibitory neurons, in a subset of ASD subjects or in certain brain regions, perhaps as a compensation mechanism for a reduction of GABA receptors (or GABAergic function) in individual inhibitory neurons^62^. However, previous studies have reported an overproduction of GABAergic inhibitory neurons in ASD iPSC-derived organoids^46^ and neural cells^47^, with the former likely resulting from increased *FOXG1* expression^46^, suggesting that an increase in inhibitory interneuron function could be due to a direct effect of some candidate genes. Another key transcription factor in GABAergic interneuron differentiation, *DLX1*, was also upregulated in *CHD8* knockout NPCs, neurons^49^, and cerebral organoids^50^. Furthermore, our study indicates that both primary and secondary ASD-affected genes may play roles in inhibitory neurogenesis and function, contributing to ASD pathogenesis. We should note that when, where and how an E/I imbalance contributes to ASD is unclear and certainly beyond the scope of the current study. Nevertheless, it is conceivable that E/I imbalance may tilt to one direction in a subset of ASD but to the other in a different subset.

Since neuronal subtype transcriptomes used in the current study were from an adult female brain^35^, and there are significant transcriptional (and structural) differences in the brain between the pre- to post-natal period, and from the teenage to adult stage^80^, it would be interesting to perform a similar EWCE study using scRNA-seq data from prenatal or fetal neurons in multiple brain regions from both sexes. Considering our findings, it is interesting to note that drugs targeting inhibitory neuron function are being developed to treat ASD^81^. Consequently, it would be valuable to study their effects in early and late developing brains, animal models, iPSC models, and in ASD subjects using brain imaging and electrophysiology to fully explore the therapeutic potential of such drugs.

Finally, we found that upregulated genes in postmortem ASD brains were enriched in microglia and astrocytes, which is consistent with original reports based on the mouse transcriptome^42,44^. This is consistent with the findings that activated microglia and astrocytosis occur in multiple brain regions of ASD patients^82,83^. However, ASD candidate themselves did not show such an enrichment in our analysis. Thus, dysregulation of neuron-glia signaling might be a secondary process in response to the initial insults elicited by the primary casual genetic variants, a testable hypothesis.

## Electronic supplementary material

Figure S1

Figure S2

Figure S3

Figure S4

Table S1

Table S2

Table S3

file with changes highlighted
