# Supplementary material for: Enriched expression of genes associated with autism spectrum disorders in human inhibitory neurons
Source: Transl Psychiatry. 2018 Jan 10;8:13. doi: 10.1038/s41398-017-0058-6 (PMC5802446; doi:10.1038/s41398-017-0058-6)
Supplement: Supplementary file 1 — Figure S1 [file 41398_2017_58_MOESM1_ESM.pdf]

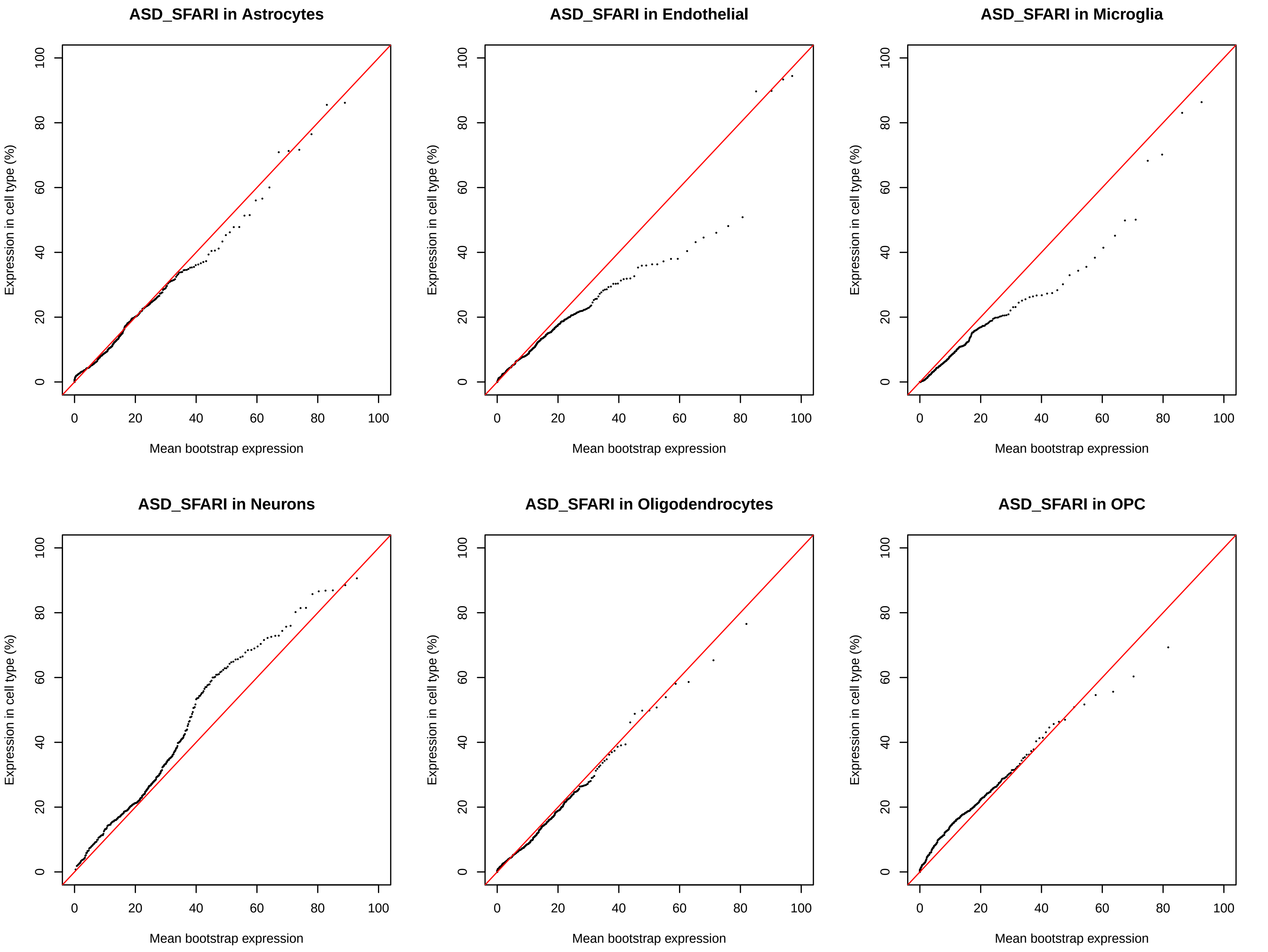

ASD_SFARI in Astrocytes
ASD_SFARI in Endothelial
ASD_SFARI in Microglia
ASD_SFARI in Neurons
ASD_SFARI in Oligodendrocytes
ASD_SFARI in OPC
Expression in cell type (%)
Mean bootstrap expression
0
20
40
60
80
100
