# Supplementary figures and images for: Enriched expression of genes associated with autism spectrum disorders in human inhibitory neurons
Source: Transl Psychiatry. 2018 Jan 10;8:13. doi: 10.1038/s41398-017-0058-6 (PMC5802446; doi:10.1038/s41398-017-0058-6)

**SFARI in In1**

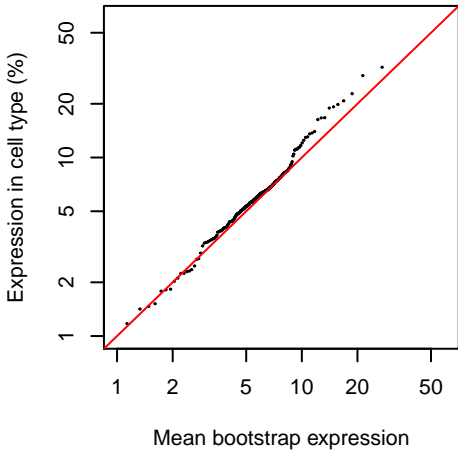

**AutismKB in In1**

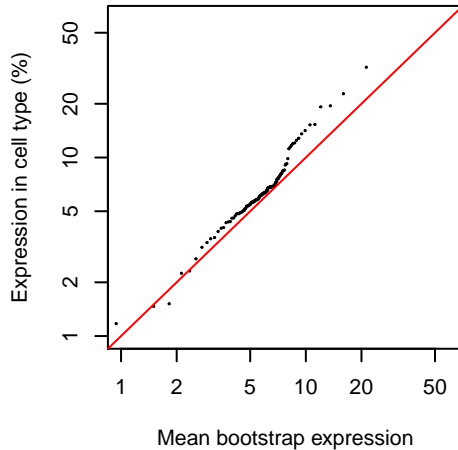

**AutismKB in In3**

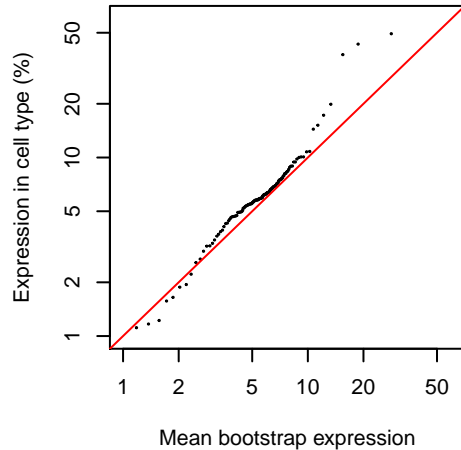

Supplement: Supplementary file 2 — Figure S2 [file 41398_2017_58_MOESM2_ESM.pdf]

**A**

### Cluster Dendrogram

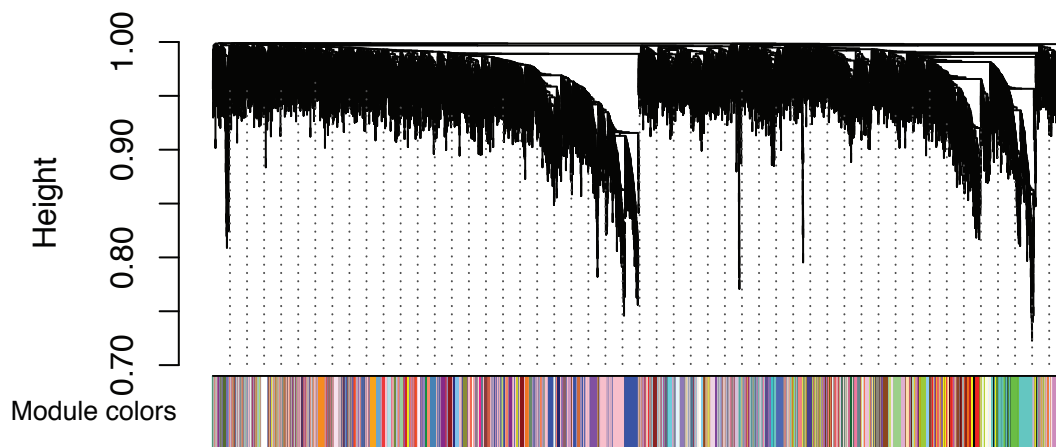**B**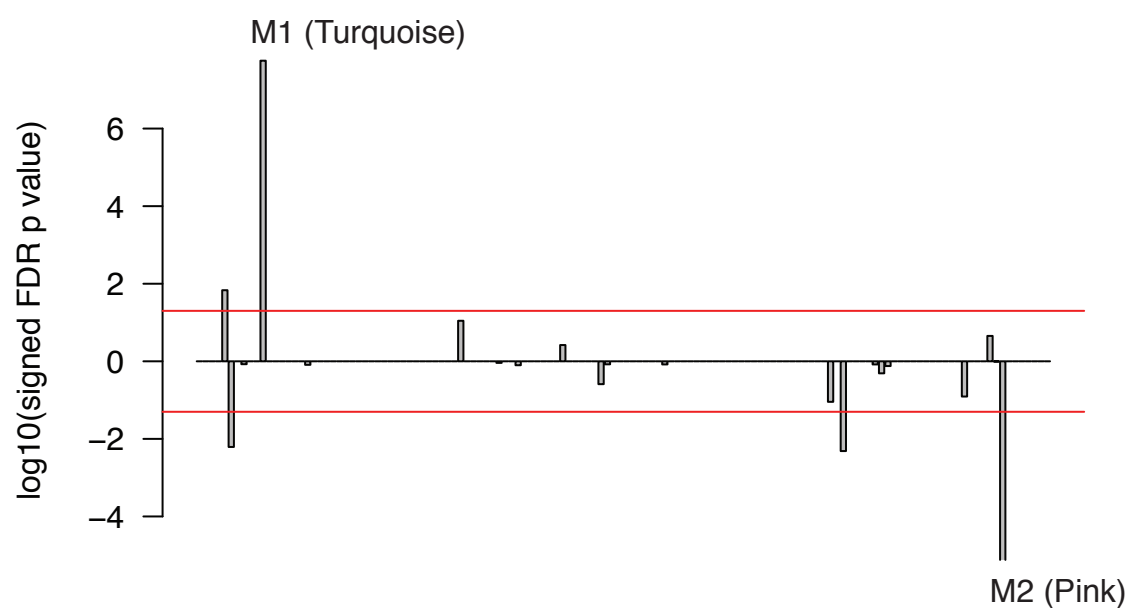**C**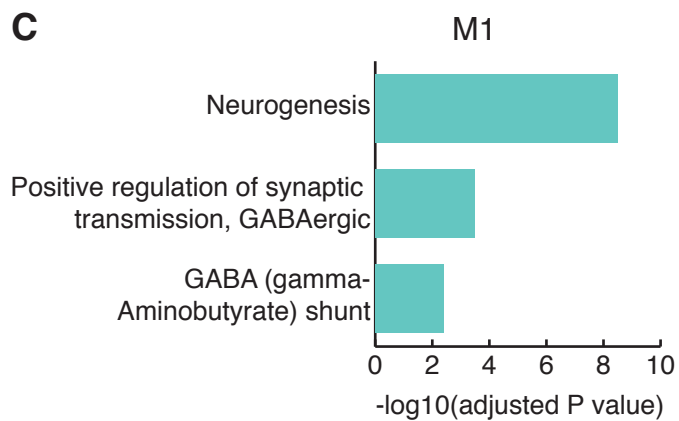**D**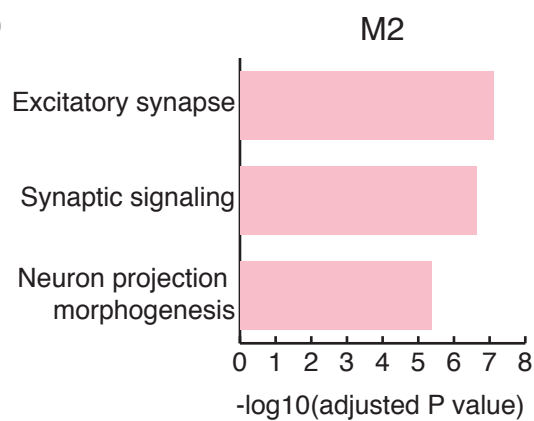

Supplement: Supplementary file 3 — Figure S3 [file 41398_2017_58_MOESM3_ESM.pdf]

## Upregulated genes in ASD samples

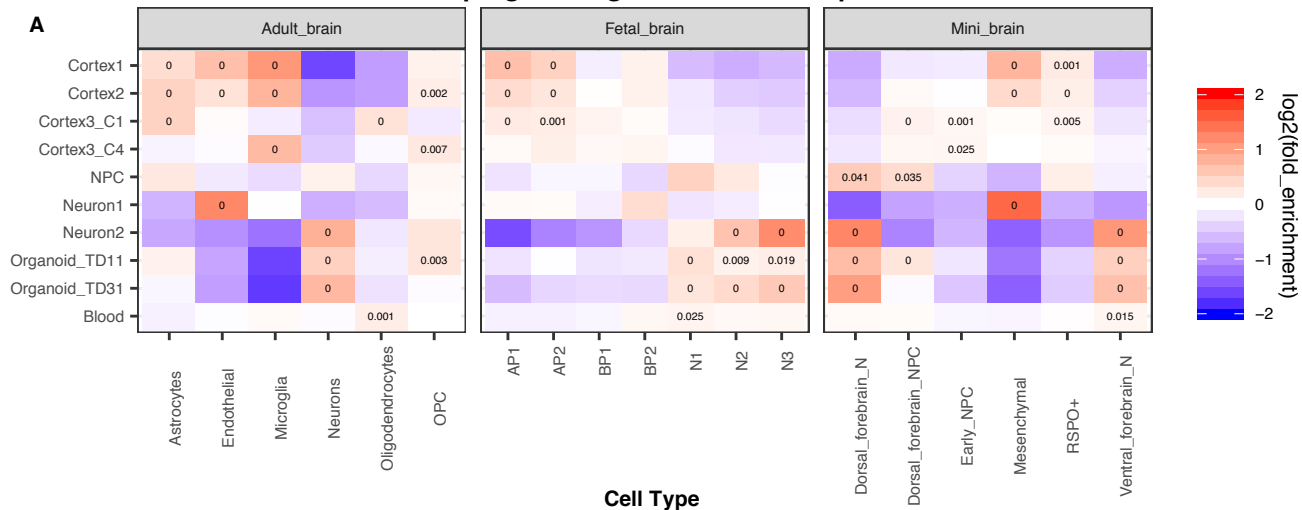

## Downregulated genes in ASD samples

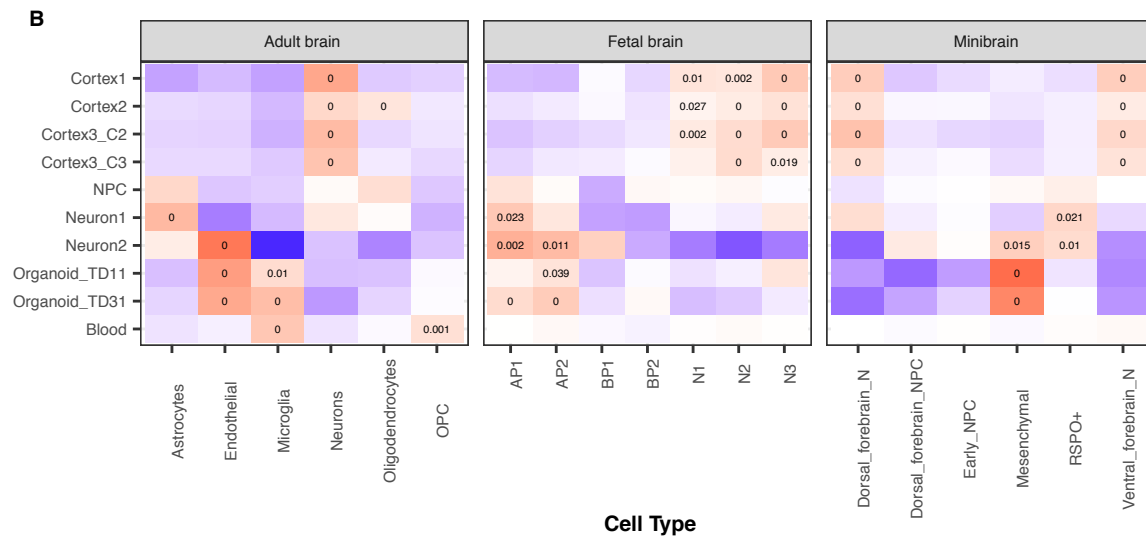

Supplement: Supplementary file 4 — Figure S4 [file 41398_2017_58_MOESM4_ESM.pdf]
